# Application of Prandtl’s Theory in the Design of an Experimental Chamber for Static Pressure Measurements

**DOI:** 10.3390/s21206849

**Published:** 2021-10-15

**Authors:** Pavla Šabacká, Vilém Neděla, Jiří Maxa, Robert Bayer

**Affiliations:** 1 Institute of Scientific Instruments of the CAS, 61264 Brno, Czech Republic; xhlava44@vutbr.cz (P.Š.); vilem@isibrno.cz (V.N.); 2 Department of Electrical and Electronic Technology, Brno University of Technology, 61100 Brno, Czech Republic; xbayer02@vut.cz

**Keywords:** ESEM, BD sensor, static probe, static pressure, mach number, differentially pumped chamber, Prandtl’s theory

## Abstract

Pumping in vacuum chambers is part of the field of environmental electron microscopy. These chambers are separated from each other by a small-diameter aperture that creates a critical flow in the supersonic flow regime. The distribution of pressure and shock waves in the path of the primary electron beam passing through the differentially pumped chamber has a large influence on the quality of the resulting microscope image. As part of this research, an experimental chamber was constructed to map supersonic flow at low pressures. The shape of this chamber was designed using mathematical–physical analyses, which served not only as a basis for the design of its geometry, but especially for the correct choice of absolute and differential pressure sensors with respect to the cryogenic temperature generated in the supersonic flow. The mathematical and physical analyses presented here map the nature of the supersonic flow with large gradients of state variables at low pressures at the continuum mechanics boundary near the region of free molecule motion in which the Environmental Electron Microscope and its differentially pumped chamber operate, which has a significant impact on the resulting sharpness of the final image obtained by the microscope. The results of this work map the flow in and behind the Laval nozzle in the experimental chamber and are the initial basis that enabled the optimization of the design of the chamber based on Prandtl’s theory for the possibility of fitting it with pressure probes in such a way that they can map the flow in and behind the Laval nozzle.

## 1. Introduction

Currently, the Department of Electrical and Electronic Engineering of the Brno University of Technology in cooperation with the Institute of Instrumentation Technology of the Academy of Sciences of the Czech Republic in Brno is conducting research on environmental electron microscopy with a focus on the field of vacuum pumped chambers, and particularly on differentially pumped chambers and chambers for samples that are separated by a small diameter aperture, which causes a critical flow in the supersonic regime ending in a shock wave. The pressure and shock wave distribution in the primary path of the electron beam passing through the differentially pumped chamber has a great influence on the resulting image quality [1,2,3]. The environmental scanning electron microscope (ESEM) is one of the most promising tools for studying plant [4,5,6] and polymer [7,8] samples using special signal electron detectors [9], which were developed based on gas flow simulations.

The experimental chamber is currently being completed. This experimental chamber has been designed to simulate the flow condition in the aperture region between the sample chamber and the differentially pumped chamber, between which there is normally a pressure difference of 100 Pa to 2000 Pa. It was necessary to perform mathematical and physical analyses because of the planned measurement of flow pressures, velocities and temperatures in that region of the supersonic flow at low pressures. The objective of this work was to perform the necessary analyses to determine the final shape of the Laval nozzle using Prandtl’s theory and to evaluate the flow condition in and behind the nozzle to determine the appropriate shape and position of the Pitot tube and temperature probe.

At the beginning of these low-pressure studies, it was necessary to compare the Monte Carlo method with the continuum mechanics method. This comparison was published in the paper Comparisons Using Methods of Continuum Mechanics and Monte Carlo at Differentially Pumped Chamber [10].

Comparison of the Monte Carlo simulation results with those obtained using ANSYS Fluent shows that both methods provide similar results [10]. On the basis of this study, which confirmed the possibility of using Ansys Fluent to map the flow at the limit of continuum mechanics, a model of the experimental chamber was designed, the shape of which allows the use of probes utilizing Prandtl’s theory.

Membrane sensors will be used to measure the pressures on the walls of the Laval nozzle and in the chambers in a configuration that will operate reliably at the expected pressures while meeting high measurement accuracy and avoiding their overloading.

## 2. Experimental Chamber

**Figure 1:**
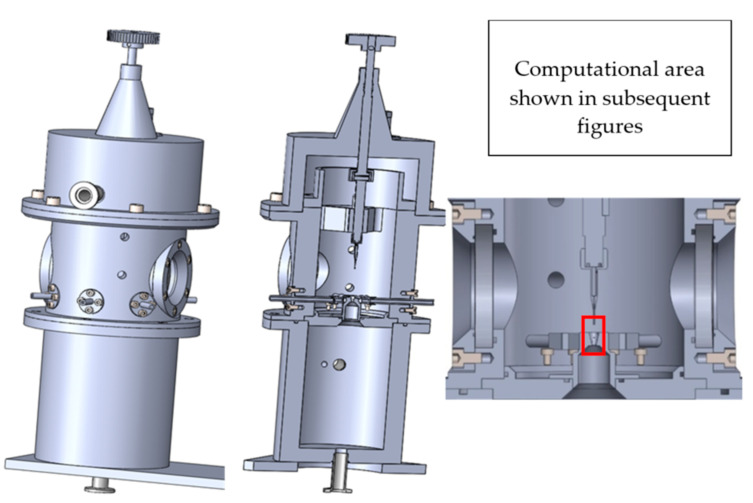
Experimental chamber.

The designed experimental chamber for mapping the pressure at the limit of continuum mechanics consists of two chambers separated by a small aperture, simulating the condition occurring during differential pumping. In the aperture, under these conditions, a critical flow is generated, manifested by a so-called nozzle clogging, beyond which a supersonic flow is generated with a low-pressure region ending in a specific shock wave [11]. The designed chamber allows the exchange of the aperture, which will make it possible to experimentally analyze different aperture shapes. It also includes a sliding mount for different types of measuring devices (Figure 1). This chamber was manufactured at the Institute of Scientific Instruments and loaned to the Department of Electrical and Electronic Technology of the Brno University of Technology, where it is currently being assembled. Finally, the chamber is also equipped with two visors through which it will be possible to observe and analyze the investigated area of flow and shock waves using the Schlieren method.

These measurements are planned in several stages in this experimental chamber:

- Measurement of the flow velocity using a Pitot tube,
- Measurement of the pressure in the primary electron beam path,
- Temperature measurements in the supersonic flow region using a thermocouple,
- Analysis of the supersonic flow by the Schlieren optical method.

For these measurements, interchangeable holders will be used for different probe mountings for pressure and temperature measurements.

Due to the difference in the diameter of the aperture (2 mm) and the experimental chamber itself (80 mm), it will not be possible to construct the Pitot tube in one piece, but the static pressure and total pressure will be measured separately.

## 3. Flow Analyzes in the Experimental Chamber

The mathematical–physics analyses of the flow in the experimental chamber in Ansys Fluent system were used as a base for its own construction [12]. The layouts for static pressure, total pressure, velocity, Mach number, and temperature were mapped in the area of the whole chamber but primarily in the area of supersonic flow [13,14]. This has a great impact on the scattering of the primary beam when passing through the Differentially pumped chamber [15,16].

**Figure 2:**
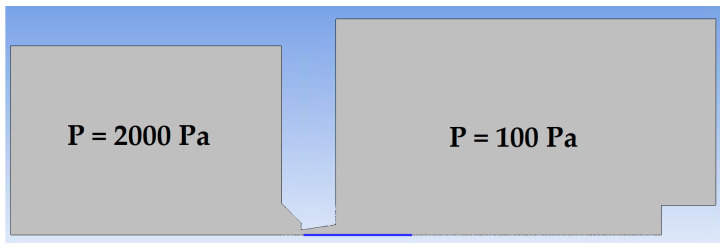
Cross-section through the experimental chamber in the aperture area.

**Figure 3:**
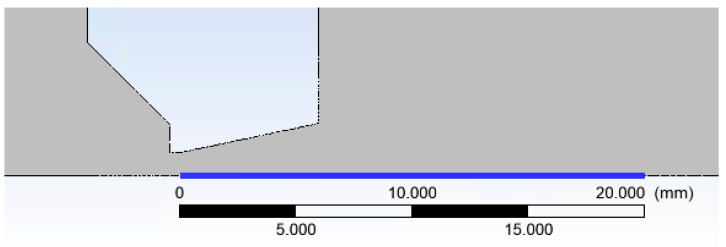
Cross-section through the experimental chamber in the aperture area (detail).

A cross-section of the experimental chamber is shown in Figure 2. An axisymmetric calculation has been performed, which allows the calculation to be accelerated due to the assumption of axisymmetry of the flow [17]. In Figure 3, the path along which other variables were calculated is marked in blue. In general, the entire chamber will be mapped for pressure and temperature, but a particular emphasis will be placed on the supersonic flow and the mapping of the nozzle and the flow behind it [18,19].

The simulation used continuum mechanics, where the Ansys Fluent system uses the Navier–Stokes equations, which are second-order partial nonlinear differential equations. These equations cover all aspects of real fluid behavior including turbulence. The given equations have been solved using the finite volume method.

Due to the compressibility of the flow and the assumption of high-pressure gradients associated with supersonic flow, a density-based fluid density solver was used. In this particular case, we used an implicit formulation where the unknown values are given by the existing values and also the unknown values of the neighboring cells. We also used the Advection Upstream Splitting Method (AUSM), which is more suitable for solving supersonic flow. For discretization, we used the second-order method.

As in this case, it is a very low-pressure environment, it is necessary to check the value of the Knudsen number to see if it is still a continuous environment.

To map the Knudsen number in a dimensionally various space, a method that has been successfully published by the FOM Institute for Plasma Physics Rijnhuizen and the Department of Applied Physics, Eindhoven University of Technology [20] was used.

The Knudsen number can be checked according to:

(1) $$Kn=\frac{\lambda }{L}$$

where *λ* is the mean free path of gas molecules and *L* is the characteristic dimension [20].

For the mean free path, the following applies:

(2) $$\lambda =\frac{kT}{\sqrt{2}\pi \delta^{2}p}$$

where *k* is the Boltzmann constant, *T* is the absolute temperature, *δ* is the gas molecule diameter and *p* is the pressure.

According to [21], the calculation of the characteristic dimension *L* as Density/Density Gradient was performed.

**Figure 4:**
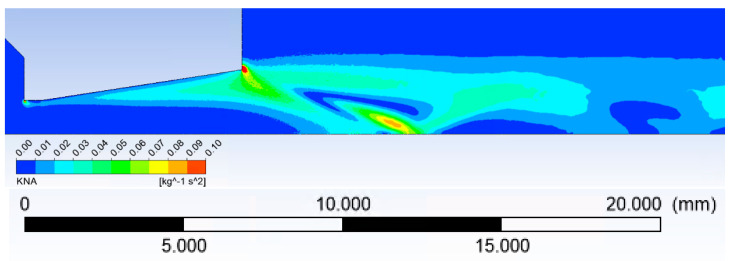
The distribution of the Knudsen number.

The distribution of Knudsen number is shown in Figure 4:

With respect to the Knudsen number, the calculation was solved in Slip Flow mode. The findings published in [21,22] were used for the first setting of the Shear stress on the wall for the Slip Flow model and its subsequent debugging.

The design principle of the experimental chamber described below is based on the experience and work of Dr. Danilatos.

As in [3], a comparison was made between the results obtained with Ansys Fluent and the results published by Dr. Danilatos in the field of thin aperture [23,24] and the results for the conical aperture. The findings published by Dr. Danilatos in [24] in the field of nozzle opening angle selection are followed in this paper in a different way, where the shape of the nozzle cone is determined by the Expansion cross-section calculation.

Dr. Danilatost often maps flow in narrow spaces that are typical for certain types of differential pumping [25]. In dimensionally small chambers, the supersonic flow has the character of an elongated cone and each additional even a smaller narrowing act as an additional aperture. Therefore, for the investigated case, the chamber behind the nozzle was designed in such a way that the supersonic flow was not affected in any way and the subsequent mathematical and physical analyses then showed no effect of even reflected shock waves on the gas flow in the path of the primary electron beam.

## 4. Static Pressure Measurement According to Prandtl’s Theory

**Figure 5:**
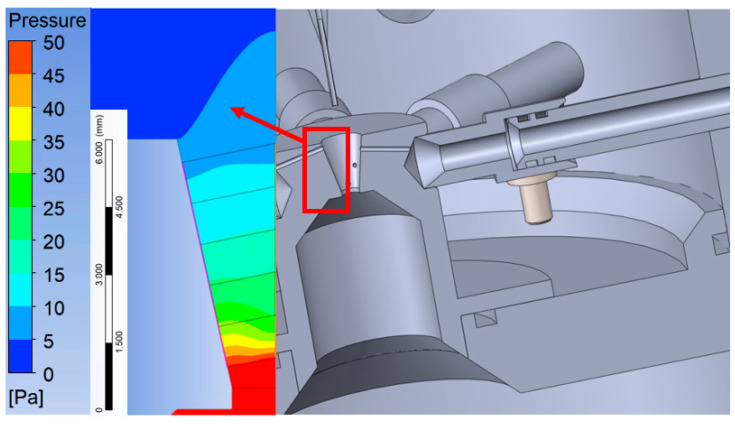
The shape of the conical Laval nozzle for Prandtl’s theory.

As already mentioned, the classical Pitot tube for static pressure measurement does not allow to capture the pressure in the nozzle, but only in the area above the nozzle, due to the small size of the chamber. Therefore, a different measurement method was used. This involves the elimination of static pressure using small holes spirally arranged around the circumference of the nozzle outlet based on Prandtl’s theory, according to which the static pressure distribution in the flow cross-section has equal values. This applies, for example, to laminar flow through a pipe. In a supersonic flow behind the nozzle throat, the gas expands in such a way that the exact shape of the nozzle can be assumed based on the calculated cross-section when applying Prandtl’s theory (Figure 5).

**Figure 6:**
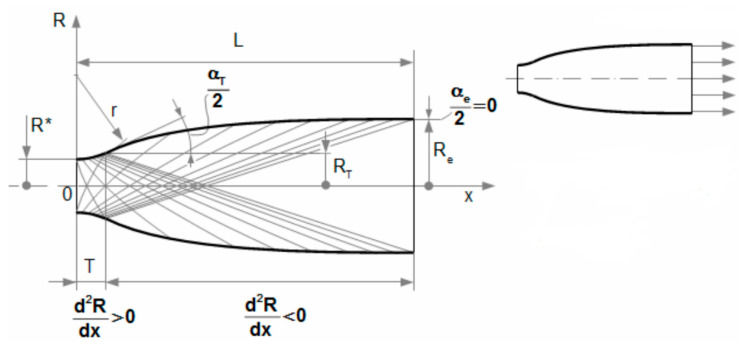
Laval nozzle—characteristic method. Reprinted from ref. [26].

**Figure 7:**
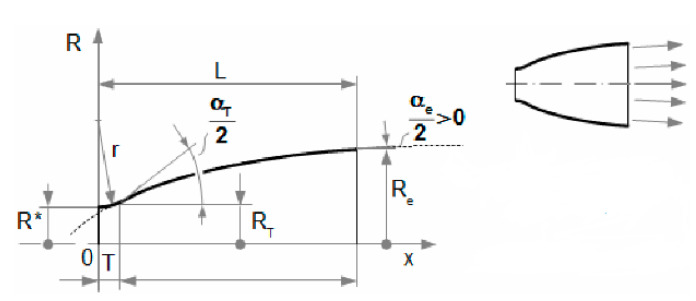
Laval nozzle—Bell nozzle. Reprinted from ref. [26].

**Figure 8:**
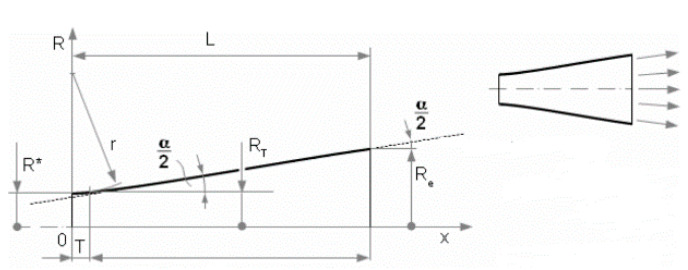
Laval nozzle—Linear shape. Reprinted from ref. [26].

The most accurate shape which copies the gas expansion is the Laval nozzle. This can be constructed in three ways: characteristic method, linear shape, or Bell nozzle (Figure 6, Figure 7 and Figure 8) [7]. Due to the production technology, the most advantageous construction is based on linear shape (Figure 8).

(3) $$r_{t}=r^{*}+r_{r}\left(1-\cos\frac{\alpha_{1}}{2}\right)$$

(4) $$t=r_{r}\cdot \sin\frac{\alpha_{1}}{2}\;$$

(5) $${\left(\frac{dr}{dx}\right)}_{t}=\tan\frac{\alpha_{1}}{2},{\left(\frac{dv}{dx}\right)}_{e}=\tan\frac{\alpha_{2}}{2}$$

(6) $$I=t+\frac{r_{e}-r_{t}}{tg\frac{\alpha }{2}}$$

The calculation of the gas expansion dimensions, which is based on the shape of the Laval nozzle, was determined based on the physics of the isentropic one-dimensional flow. This leads to relationships that determine the relationship of pressures, densities, velocities, and Mach numbers between the inlet area of the nozzle, within the nozzle, and the calculated gas expansion cross-section behind the nozzle [27].

For isentropic flow, the following applies:

(7) $$\frac{v_{v}}{v_{kr}}={\left[\frac{\left(\varkappa +1\right)M^{2}}{2+\left(\varkappa -1\right)M^{2}}\right]}^{\frac{1}{2}}\;$$

(8) $$\frac{v_{v}}{v_{o}}={\left[\frac{2}{2+\left(\varkappa -1\right)M^{2}}\right]}^{\frac{1}{2}}$$

(9) $$\frac{T_{v}}{T_{o}}=\frac{2}{2+\left(\varkappa -1\right)M^{2}}$$

(10) $$\frac{p_{v}}{p_{o}}={\left[\frac{2}{2+\left(\varkappa -1\right)M^{2}}\right]}^{\frac{\varkappa }{\varkappa -1}}$$

(11) $$\frac{\rho_{v}}{\rho_{o}}={\left[\frac{2}{2+\left(\varkappa -1\right)M^{2}}\right]}^{\frac{1}{\varkappa -1}}$$

(12) $$\frac{\rho_{v}}{\rho_{kr}}=\frac{A_{kr}}{A}=M{\left[\frac{\varkappa +1}{2+\left(\varkappa -1\right)M^{2}}\right]}^{\frac{1}{2}\frac{\varkappa +1}{\varkappa -1}}$$

where *p*_0_ is the input pressure, *p_v_* is the output pressure, *T*_0_ is the input temperature, *T_v_* is the output temperature, *v*_0_ is the input velocity, *v_v_* is the output velocity, *v_kr_* is the critical velocity, *ρ*_0_ is the input density, *ρ_v_* is the output density, *M* is the Mach number, ϰ is the gas constant = 1.14, *A* is the computational cross-section and *A_kr_* is the critical cross-section.

**Figure 9:**
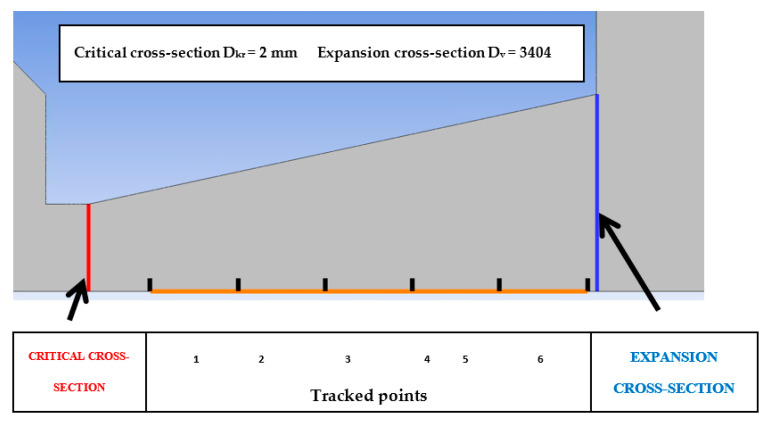
Measured points.

This computational cross-section (*A*) of the Laval nozzle was determined based on the above calculations. At these six points (Figure 9), the temperature and total pressure will be measured using probes placed on the axis, and according to Prandtl’s theory, the static pressure will be captured from the walls where the pressure distribution is assumed to be the same in both the projection and the axis.

The pressure ratio *p_o_* = 2000 Pa and *p_v_* = 100 Pa are based on the given relations and the results are shown in Table 1:

In addition, the speed of sound at the input in the given environment is determined from relation 8 and equals 346.7 ms^−1^:

(13) $$v_{0}=\sqrt{XRT_{0}}\;$$

where *R* is the universal gas constant and *T*_0_ = 297.15 K.

In Table 1, the ratio of *ρ_v_/ρ*_0_ = 0.0617. Then, it is possible to determine the value of output density *ρ_v_* = 0.00276 kg·m^−3^.

After the mathematical and physical analyses in Ansys Fluent, a back check was performed according to the physics of one-dimensional flow for the calculated cross-section. The values obtained theoretically were compared with the values obtained using Ansys Fluent and the measurement errors are minimal in this case.

The values of *v_v_*, *ρ*_0_, *ρ_v_* and *T_v_* were used as control values for the results obtained with Ansys Fluent (Table 2).

Then, the size of the nozzle opening or the so-called computational cross-section can be determined, where Prandtl’s theory applies, so that the static pressure is the same throughout the cross-section.

For a given density ratio *ρ_v_*/*ρ_kr_*, which is shown in Table 1, the following applies:

(14) $$\frac{\rho_{v}}{\rho_{kr}}=\frac{A_{kr}}{A_{v}}=0.3453$$

For the selected hole diameter *D_kr_* = 2 mm, the value *A_kr_* = 3.14 mm^2^. From relation 10, the calculated cross-section is equal to *A_v_* = 9.1 mm^2^, and therefore *D_v_* = 3.4 mm. The angle of 12° was determined according to [29].

The results of Dr. Danilatos [10] were used to analyze the location of the Mach disk under the given low-pressure conditions:

(15) $$z_{M}=0.67D_{kr}\sqrt{\frac{P_{0}}{P_{1}}}=6\;\mathrm{mm}\;$$

**Figure 10:**
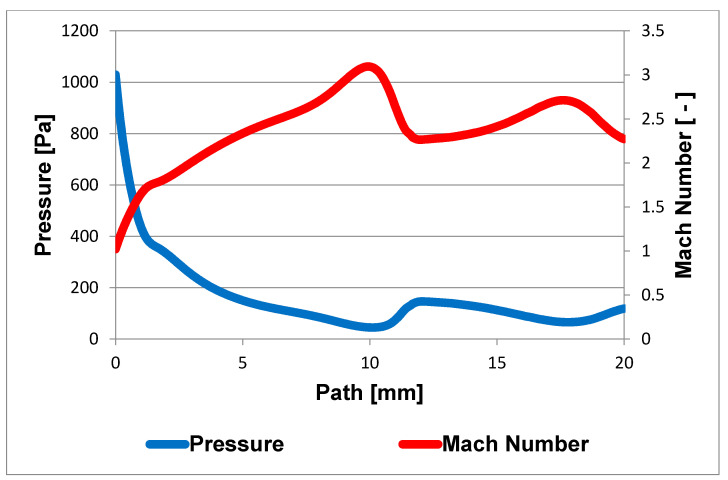
Pressure and Mach number on the path shown in Figure 3.

With a nozzle length of 5.941 mm, the distance of the Mach disk is approximately 12 mm from the critical cross-section. Subsequent analysis of the ANSYS Fluent results showed the same Mach disk distance (Figure 10), with experience with Mach disk placement also drawn from [30].

**Figure 11:**
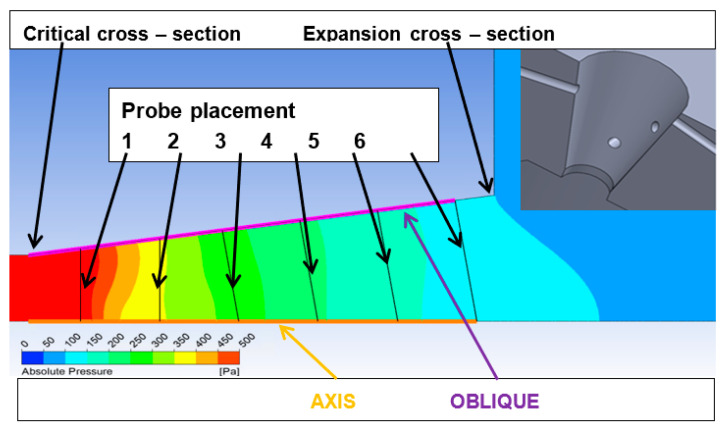
Designed linear nozzle with measuring points for probe placement.

Figure 10 shows the formation of two Mach disks at distances of 12 mm and 19 mm from the critical section. This will result in a decrease in flow velocity and an increase in pressure compared to the region beyond the critical section or behind the Mach disk, behind which a region of low pressure and therefore high flow velocity is created due to particle entrainment by the ambient flow. From Figure 11, it can be seen that the measured points were selected according to Prandtl’s theory, according to which the holes were spirally arranged along the nozzle exit cone, and whose perpendicular projection points to these selected points. At the same time, according to the first calculations of the static pressure distribution in the nozzle, it can be seen that Prandtl’s theory is correctly applied. However, in the region of the second measured point, the pressure distribution is slightly rotated precisely because of the chosen technologically simpler linear shape of the nozzle.

**Figure 12:**
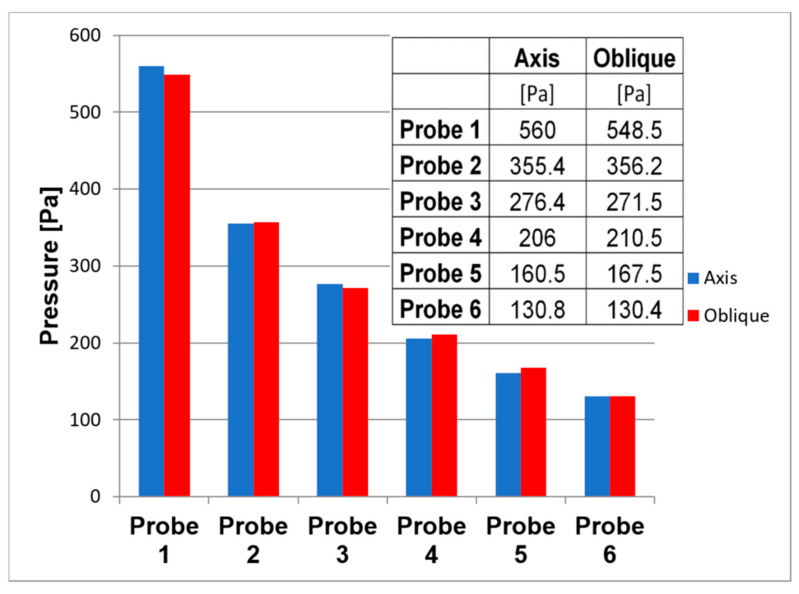
Comparison of static pressure on the axis and oblique surface of the nozzle according to Prandtl’s theory.

Comparing the pressure profile, it is clear from Figure 12 that the differences are minimal. However, at points 1 and 2, the pressure profile is rotated due to the perpendicularity of the holes to the axis to simplify the shape of the Laval nozzle.

## 5. Use of a Membrane Pressure Difference Sensor for Low Pressures Measurements

**Figure 13:**
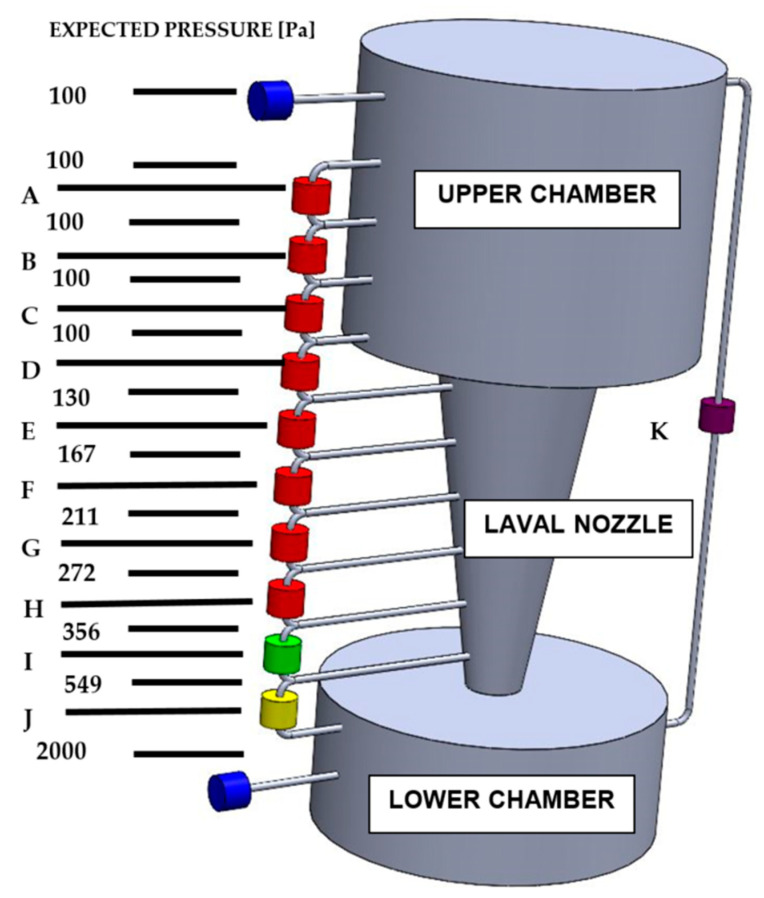
Probe placement on the experimental chamber.

Using the mathematical and physical analyses above, it was found that the pressure distribution in the two chambers separated by the Laval nozzle corresponds to the pressure distribution in the nozzle itself. As a result, pressure differences between the planned pressure measurement points were also found [31]. Figure 13 shows a diagram of the distribution of measurement points in the experimental chamber with the pressures at the probe outlets marked.

From these predicted measured pressures, the expected pressure difference at the pressure probes is calculated, see Table 3.

Figure 13 is for illustrative purposes only. For better illustration, the Laval nozzle separating the two chambers is shown here enlarged disproportionately to its surroundings. The correct ratio of the sizes of the Upper and Lower Chambers and the Laval nozzle can be seen in Figure 1 and Figure 2. The distance between points A and H (the two counterpoints of the probes with the finest resolution) is 68 mm. Due to the above-mentioned illustrative nature of Figure 13, it should be pointed out that the spacing of the probes E, F, G and H, which map the pressure distribution in the Laval nozzle, are 1 mm, while the spacing of the probes A, B, C and D, which map the pressure distribution in the upper chamber, are 20 mm.

The upper and lower chambers are measured with absolute manometers to measure the absolute pressure in both chambers. However, between the two chambers, only differential pressure gauges are connected in series.

The selected differential pressure sensor is a DPS 300 from BD Sensors. The DPS+ differential pressure sensor was developed for the measurement of dry non-aggressive gases and compressed air. The basic element of the DPS+ is a piezoresistive pressure sensor with temperature compensation, which is able to operate for a very long time without any maintenance. The Robust design also allows deployment in the laboratory and industrial environments. The DPS+ pressure sensor is characterized by excellent long-term stability, linearity and repeatability. Therefore, it is suitable for our experiment [32,33].

Given the values shown in Table 3, the following variants of the PCB 300 probes will be used:

- For probe J: differential pressure value 4000 Pa.
- For probe I: differential pressure value 400 Pa.
- For probes A–H: differential pressure value 160 Pa.

The measurement error for the probes is the following:

- For the range below 600 Pa ± 0.5% of the range.
- For the range above 600 Pa ± 1% of the range.

At key points on the surface of the Laval nozzle where the pressure difference between the sensors is 30 Pa, the measurement error will be 0.8 Pa.

The principle of measurement will be that the absolute gauges will read the absolute pressures in the upper and lower chambers and monitor the error that occurs in the differential probe series.

Once the desired pressure ratio of 2000 Pa in the upper chamber and 100 Pa in the lower chamber is reached, the differential sensors will be connected as they cannot be connected when pumping from atmospheric pressure because they would be damaged by overloading. Once the differential gauges are connected, the differential pressures will be measured.

At the same time, the pressure difference between the two chambers shall be measured using probe K and the error of the difference between the absolute pressures measured by the absolute gauges and the pressure difference measured by the differential gauge between the two chambers shall be evaluated.

At the same time, the error of the sum of the individual differences measured by the differential gauges in series between the two chambers shall be evaluated.

The results will be compared with those obtained by mathematical and physical analysis from Ansys Fluent.

## 6. Conclusions

The paper is a follow-up to the comparative analyses carried out at the Institute of Electrical and Electronic Engineering FEEC BUT in cooperation with the Institute of Scientific Instruments of the CAS, v.v.i., where the possibility of using the Ansys Fluent system using continuum mechanics is compared with the analyses carried out in the Monte Carlo system of Dr. Danilatos. On the basis of these mathematical and physical analyses from Ansys Fluent, the design of an experimental chamber for supersonic flow at low pressures at the limit of continuum mechanics was realized. This experimental chamber will simulate the differential pumping conditions in the aperture region between the sample chamber and the differentially pumped chamber in an environmental scanning electron microscope. Since this is a very small space where conventional probes could not be placed, it was necessary to determine the final shape of the Laval nozzle according to Prandtl’s theory by mathematical and physical analyses to measure unbiased pressure readings in the flow axis and from the nozzle wall.

The results of the mathematical–physical analyses applying Prandtl’s theory, described in this paper, allowed the necessary corrections to be made to the geometry of the Laval nozzle inside the pumped chamber to measure the static pressure inside this nozzle by simplifying the shape of its opening while maintaining its properties.

The results of the analyses were also the basis for the selection of the type of absolute and differential pressure probes and temperature probes related to the cryogenic regime.

Without the information obtained from these analyses, several time- and cost-consuming experiments would have been needed.

## Figures and Tables

**Table 1:** Calculated values in critical input cross-section [28].

Mach Number	Output Velocity/Critical Velocity	Output Velocity/Input Velocity	Output Temperature/Input Temperature	Output Pressure/Input Pressure	Output Density/Input Density	Output Density/Critical Density
*M_v_*	*v_v_/v_kr_*	*v_v_/v*_0_	*T_v_/T*_0_	*p_v_/p*_0_	*ρ_v_/ρ*_0_	*ρ_v_/ρ_kr_*
2.6	1.8571	0.6521	0.4252	0.05	0.1179	0.3453

**Table 2:** Results of comparison of Ansys values with one-dimensional flow theory.

	Theoretical Value	Ansys Fluent Value
Mach number (-)	2.6	2.598
Density (kg·m^−3^)	0.00276	0.00265
Velocity (m·s^−1^)	585.8	582
Temperature (°C)	126.3	126.8

**Table 3:** The expected pressure difference in the pressure probes.

**PROBE**	A	B	C	D	E	F	G	H	I	J
**EXPECTED PRESSURE DIFFERENCE (Pa)**	0–5	0–5	0–5	30	37	44	61	84	193	1451

## Data Availability

The data presented in this study are available on request from the corresponding author.
